# Response inhibition training as an intervention to modify liking and wanting for foods based on energy density: a proof of concept study

**DOI:** 10.1007/s10865-023-00453-3

**Published:** 2023-10-09

**Authors:** Halim Moore, Melanie J. White, Graham Finlayson, Neil King

**Affiliations:** 1 School of Exercise and Nutrition Sciences, Queensland University of Technology, 60 Musk Avenue, Kelvin Grove, 4059 Australia; 2 School of Psychology and Counselling, Queensland University of Technology, Kelvin Grove, 4059 Australia; 3 School of Psychology, University of Leeds, Leeds, LS2 9JT UK

**Keywords:** Response inhibition, Cognitive training, Food preference, Mobile application, Energy density, Food reward

## Abstract

The ubiquity of energy-dense, processed foods has been implicated as a salient feature of the modern ‘obesogenic’ environment. Cognitive strategies, such as response inhibition training, have been demonstrated to reduce the hedonic value of such foods in previous studies. However, this effect has generally been inconsistent or heterogenous, depending on the outcome measure, characteristics of the sample, and the specificity of food stimuli. Characterising the extent of generalised effects may help define the application of this type of intervention in natural settings. A repeated-measures, proof-of-concept study, using mobile app-based response inhibition training (RIT) versus a control app-based activity (*N* = 25), was undertaken to establish the valid application of a food reward measure to assess intervention efficacy. Liking (i.e., affect) and wanting (i.e., motivation) for food stimuli categorised by energy density were taken concurrently pre- and post-training. A statistically significant reduction in explicit liking, but not implicit wanting, for foods irrespective of their energy density was observed during the RIT app-based training session relative to the control (*p* = .041, *ηp*^*2*^ = .16). However, effect sizes associated with devaluation of energy-dense relative to low calorie food stimuli, although non-significant, were higher when measured as implicitly wanting (*p* = .098, *ηp*^*2*^ = .11) than explicit liking (*p* = .756, *ηp*^*2*^ = .00). Trends in explicit stimulus evaluations were empirically discordant from implicit evaluations for low calorie foods in particular. Additional research is needed to investigate whether these trends are reproducible with larger samples, trained and novel food stimuli in outcome measures, and more comprehensive training protocols.

**Supplementary Information:**

The online version contains supplementary material available at 10.1007/s10865-023-00453-3.

## Background and study aims

Cognitive bias modification techniques have been used to promote and sustain healthier food preferences, especially in individuals susceptible to weight gain and excess food consumption (see Allom et al., 2016; Aulbach et al., 2019; Jones et al., 2016; Turton et al., 2016; Yang et al., 2022 for reviews). Go/No-Go, as a form of inhibitory control training purporting to modify food-specific cognitive biases, is an associative learning protocol that pairs a clear auditory or visual signal for a prescribed behavioural response with a corresponding salient stimulus (Verbruggen et al., 2014). When implemented as a dietary intervention, the Go/No-Go training paradigm, and similarly designed tasks such as Stop-Signal, conventionally pair a salient signal denoting a stop action in the case of the latter, or response inhibition in the case of the former, with a proscribed food cue (e.g., energy-dense, hyperpalatable, ultra-processed, high fat-high sugar, etc.).

The extant body of evidence suggests that response inhibition training (RIT), an iteration of the Go/No-Go, has demonstrated modest efficacy in modulating hedonic liking, food choice, and even weight (Jones et al., 2018). The most reliable effect has been shown through measurement of explicit evaluations of food cues, usually by ratings of liking along a visual analogue scale (Adams et al., 2021; Chen et al., 2018; Lawrence et al., 2015a, 2015b; Najberg et al., 2021; Veling et al., 2013a, 2013b; Yang et al., 2021, 2022). However, RIT has also been associated with changes in simulated food choice, self-selected portion sizes, and relative reinforcing value for palatable, energy-dense foods (Chen et al., 2019; Houben & Geisen, 2018; Porter et al., 2018; Stice et al., 2017; Van Koningsbruggen et al., 2014; Veling et al., 2021). Additionally, efficacy has been observed in lab-based food intake (Houben, 2011; Houben & Jansen, 2015; Oomen et al., 2018) and even weight loss (Lawrence et al., 2015b; Veling et al., 2014). Pooled effect sizes for food intake (Aulbach et al., 2019) and evaluation (Yang et al., 2022) tend to range between Hedges *g* = 0.25–0.38 for food-specific Go/No-Go tasks specifically, higher relative to alternative bias modification tasks such as the stop-signal task (Hedges *g* = 0.11–0.14) and approach-avoidance task (Hedges *g* = 0.09).

The findings from previous RIT trials, indicative of both conditional efficacy and methodological heterogeneity, has highlighted the need to elucidate the nature of the RIT effect on food evaluation, including neurobehavioural mechanisms of action (Carbine & Larson, 2019; see Veling et al., 2017 for a discussion). Theoretical frameworks of addiction may provide insight when interpreting or contextualising the food devaluation effect observed in previous RIT studies. Berridge and Robinson (2016) have posited, after extensive research mainly in animal models, that the dopamine depletion characteristic of those demonstrating behavioural addictions for a reward-eliciting stimulus was reliably associated with an inverse augmentation of what they refer to as ‘incentive salience’, also known as ‘wanting’ more conventionally (also see Morales & Berridge, 2020 for a review). By contrast, affective response associated with consumption of the reward, or ‘liking’, remained relatively stable. Evidence to date has generally supported the hypothesis that neural pathways governing liking and wanting interact, albeit able to operate independently under certain conditions (de Araujo et al., 2020; Roefs et al., 2018; Volkow et al., 2017). Although there is some evidence to suggest that RIT can modulate motivational salience of palatable foods (e.g., Houben & Giesen, 2018; Stice et al., 2017), measures of liking and wanting are rarely measured concurrently in RIT trials. Thus, it is not certain to what degree both appetitive facets are sensitive to RIT effects, especially in varying conditions of food stimulus-specificity. Indeed, the meta-analysis by Yang and colleagues (2022) demonstrated that devaluation effects are primarily observed with ‘trained’ relative to novel food stimuli, although this difference was not statistically significant.

Therefore, the present proof-of-concept study primarily aimed to establish the feasibility of applying a nuanced framework based on Incentive Sensitisation Theory when testing the efficacy of a food-specific RIT intervention purporting to modulate appetite and food preferences. Another salient aim was to investigate whether the generalisation of RIT devaluation effects may occur as implicit wanting and/or explicit liking when food stimuli share conspicuous nutritional properties (e.g., energy density, ultra-processed, high palatability). Evidence to date suggests that effect sizes associated with devaluation of novel food stimuli are relatively smaller than those included in the RIT task (Yang et al., 2022). However, these comparisons are predominately based on measures of explicit liking, thus it may be interesting to reproduce this observation using other modalities of measuring food reward. Given the multidimensional nature of food-specific impulsivity (Van der Laan et al., 2016), the final aim was to explore to what extent different dimensions of food-specific and general trait impulsivity were associated with food reward at baseline as a method to support the suitability of this outcome measure to evaluate RIT intervention efficacy. Ultimately, trends in liking and wanting for visual food stimuli based on energy density and palatability were investigated concurrently as a method of observing coherence after completion of app-based RIT relative to a control comparison.

## Methods

### Design and participants

This study utilised a 2-way crossover design to test the feasibility of this nuanced approach of evaluating cognitive-oriented dietary intervention efficacy in a controlled trial. Participants completed both mobile app-based activities (intervention versus control app-based training). Time served as a within-subjects factor (baseline versus post-app training). Mobile app-based activity order was counterbalanced across participants using alternating allocation.^1^ To be eligible for participation, individuals had to confirm that they a. had never been diagnosed with an eating disorder; b. were not taking any recreational or prescribed drugs that significantly affected bodyweight and/or appetite; c. did not smoke cigarettes heavily and habitually (i.e., > 5 per day); d. had not experienced any significant weight changes in the last 3 months (i.e., greater than 10% of original weight); e. were not actively trying to lose weight or recently enrolled in a weight loss programme; and, f. currently 18 years of age or older.

As a feasibility trial, no sample size calculation was performed a priori*.* However, a one-tailed post-hoc analysis (Faul et al., 2007) indicated that 33% power was achieved with the final sample (*N* = 25) to detect a small to medium effect size of *d*_*z*_ = 0.25 from a comparison of dependent means based on a pooled estimate from a recent meta-analysis (Yang et al., 2022). Therefore, particular caution should be exercised when interpreting inferential statistics such as *p*-values, confidence limits, and effect sizes, whether statistically significant or non-significant, described in the present study.

### Materials and measures

#### Food reward and preference—leeds food preference questionnaire (LFPQ)

The Leeds Food Preference Questionnaire (LFPQ) was used to assess food preference and hedonics. The LFPQ consists of two separate tasks designed to operationalise corresponding distinct facets of food reward discretely: hedonic liking and motivational salience (Finlayson et al., 2007a, 2007b, 2011). This tool has been established in several empirical studies to assess food reward in both healthy weight and overweight individuals (e.g., Dalton & Finlayson, 2014; French et al., 2014; Griffioen-Roose et al., 2010, 2011). Food images are categorised dichotomously based on two nutritional and sensory qualities: energy density (high or low fat) and taste (sweet or savoury). The first task assessed explicit liking and wanting for each food image independently by asking the respondent how pleasant it would be to taste, and how much they want, the food, respectively, by 100-mm visual analogue scale.

The second task measured frequency of choice and implicit wanting by presenting distinct food pairs belonging to opposing categories and requesting respondents to choose between them as quickly and accurately as possible. Implicit wanting was operationalised using a frequency-weighted algorithm (FWA), which adjusted the standardised reaction time scores for the frequency of selection (and non-selection) within each food category. Reaction time was covertly measured when respondents indicated their choice during each trial. Thus, a higher implicit wanting score indicates a more rapid preference for a particular food category relative to its reciprocal. Mean scores from sweet and savoury foods within each fat category were averaged to determine scores for high fat and low fat foods as a comparison of foods based on energy density. Additionally, fat bias scores were calculated to indicate a participant’s predilection for high fat foods relative to low fat foods to reduce complexity in descriptive analyses. For a more detailed description of the LFPQ and its psychometric properties, see the review by Oustric and colleagues (2020).

#### RIT intervention and control tasks

The RIT intervention was delivered by the mobile app ‘FoodTrainer’, designed and produced by University of Exeter (2017) based on a Go/No-Go training paradigm where approaches to energy-dense, processed foods are inhibited whilst approaches to ‘healthy’ foods are facilitated. Examples of ‘healthy’ food from the task included predominantly fruits, vegetables, and unrefined grains. ‘Unhealthy’ foods typically consisted of energy-dense, ultra-processed foods greater than 4 kcal/g such as crisps chocolate, and other discretionary items. There was a consistent 100% ‘Go’ and 100% ‘No-Go’ task contingency for these healthy and energy-dense food stimuli, respectively. Participants completed 9 blocks in total which spanned 12 min, a session duration approximate to previous RIT studies (e.g., Adams et al., 2017; Houben, 2011; Houben & Jansen, 2015; Lawrence et al., 2015b; Veling et al., 2011, 2013a, 2013b). For a more detailed description of the RIT protocol embedded in this mobile app, see the study conducted by Aulbach and colleagues (2021).

The control activity chosen for this study was the mobile app ‘FruitNinja’ (Halfbrick Studios, 2019). This game differed from the RIT intervention in that there were depictions of ‘healthy’ food images but no energy-dense, processed foods. The gameplay mimicked a Go/No-Go training paradigm by facilitating approaches to a discrete category of desirable food stimuli (i.e., fruit) whilst inhibiting responses when exposed to another, undesirable category. Notably, the stimulus type associated with response inhibition was a non-food item, as opposed to an energy-dense food. Thereby, cognitive effort, food cue exposure, general response inhibition, and approach facilitation to ‘healthy’ foods were ostensibly held constant across sessions.

#### Food-specific and general trait impulsivity

Assessment of food-related and general trait impulsivity were completed by the participant at baseline. The specific scales used in this study are detailed below.

##### Adult eating behaviour questionnaire—food responsiveness subscale

The Adult Eating Behaviour Questionnaire (AEBQ) is a validated self-report questionnaire designed to assess an adult population on similar appetitive traits that have been associated with susceptibility to overconsumption and weight gain in children (Hunot et al., 2016). Only the 4 items belonging to the factor Food Responsiveness were included in this study given its relevance to food cue reactivity. Items are rated on a 5-point Likert. Food Responsiveness has been found to have acceptable internal reliability (α = 0.75) and good test–retest reliability (*r* = 0.87). However, in the current sample, the internal reliability was slightly lower than the acceptable threshold (ω = 0.64).

##### Three-factor eating questionnaire (Revised-18)

Salient aspects of regular eating behaviour and cognition were assessed with the revised Three-Factor Eating Questionnaire (TFEQ-R18; Karlsson et al., 2000). This validated questionnaire contains 18 items clustering around 3 dimensions: Cognitive Restraint, Uncontrolled Eating, and Emotional Eating. Items measure the extent to which a statement is true for the participant, or frequency of a relevant experience/behaviour, on a 4-point Likert scale. The internal reliability in the present sample was good for Emotional Eating and Uncontrolled Eating subscales (ω = 0.88 and ω = 0.79, respectively) and acceptable for Cognitive Restraint (ω = 0.73).

##### The Barratt impulsiveness scale-11

General trait impulsivity was evaluated using the revised Barratt Impulsiveness Scale (BIS-11; Patton et al., 1995). This validated self-report measure contains 30 items that address three separate components of general impulsivity: Attentional, Motor, and Non-planning. Participants rated the frequency by which they act or think in a certain way on a 4-point Likert scale. Internal reliability for Attentional and Non-planning subscales was acceptable (ω = 0.73 and ω = 0.72, respectively), but poor for the Motor subscale (ω = 0.62) in the current sample.

#### Subjective appetite and mood

Subjective evaluations of state appetite and mood were assessed by electronic 100-mm visual analogue scale (Flint et al., 2000; Gibbons et al., 2014). Evaluations consisted of four appetite-related and two mood-related questions which were presented to the participant sequentially. Appetite-related variables consisted of subjective hunger, satiety, desire to eat, and thirst. Mood questions were based on current feelings of contentedness and alertness. Appetite and mood were assessed at the beginning of each lab session to account for state appetite as a potential confounding factor.

#### Objective and subjective mobile application rating

The user experience during the RIT app-based task was assessed by the user Mobile Application Rating Scale (uMARS; Stoyanov et al., 2016). The questionnaire consisted of 19 items that evaluate apps based on four objective domains of quality: engagement, functionality, aesthetics, and information. Additionally, there are 4 items devoted to the participant’s subjective ratings of the app overall. Items are rated on a 5-point Likert scale, with subscale scores representing the mean of all item scores belonging to it. An overall objective quality score was derived from the mean of the four objective subscale scores. Finally, an overall Subjective Quality score was derived from the mean of the four items belonging to this subscale. Internal consistency for Engagement, Aesthetics, and Information domains was good (ω = 0.82, ω = 0.81, and ω = 0.90, respectively) and acceptable for the Functionality domain (ω = 0.74) in this sample. The internal reliability of the Subjective Quality subscale was also good (ω = 0.89).

### Procedure

All eligible persons were invited to a lab facility for the baseline session. Prior to all sessions, participants were reminded to refrain from any food intake for a minimum of 3 h beforehand to standardise appetite across sessions. Individuals who were eligible provided their informed consent before commencing the baseline session, assigned to a particular app-based activity sequence, and completed baseline measures of subjective appetite, food preference, and trait impulsivity questionnaires. Participants were then invited for a return visit to the lab for the first session with the mobile app-based activity, which was either the intervention or the control depending on sequence allocation. Each session commenced with participants rating their baseline subjective appetite. Time of engagement was held constant across sessions. The same smartphone was provided to each participant during all sessions to standardise screen size, resolution, and brightness during game play.

After the allotted time with each app-based activity, participants were then asked to complete the food preference assessment followed by ratings of app quality and their experience using it. The second session undertaking an app-based activity was identical to the first except the opposite app-based activity was completed. A washout period between sessions was designated at a minimum of three days to mitigate both practice effects with regard to the food preference outcome measure and carryover effects from each app-based activity. For each participant, the time of day for all sessions was maintained within 30 min of each other, which is best practice in repeated measures designs due to the circadian influences on appetite (Gibbons et al., 2014).

### Statistical analyses

2 (intervention versus control) × 2 (high fat versus low fat) repeated measures ANOVAs were conducted with delta scores (i.e., post – baseline) of explicit and implicit food reward metrics to compare app-based training activities for each relevant category of food based on energy density without additional complexity from higher-order interactions. Specifically, these were explicit liking, explicit wanting, frequency of choice, and implicit wanting. Additionally, mean differences with confidence intervals (CI) ranging from 80–95% were computed using one-way ANCOVAs to compare changes in fat bias scores (i.e., high fat relative to low fat) after each app-based training session. As a proof-of-concept or feasibility experiment with insufficient power to detect a small-to-medium sized significant effect of RIT, descriptive statistics with a range of reliability estimations were included in addition to inferential testing of intervention effects in line with recommendations from Lee and colleagues (2014). Means for subjective appetite sensations during each session (i.e. hunger, fullness, and desire to eat) were compared to check for potential confounding. Where assumption of sphericity was violated, Greenhouse–Geisser corrections were used. Pairwise comparisons were based on estimated marginal means with the Bonferroni adjustment. Finally, exploratory Pearson’s correlations with 95% CI were conducted to investigate bivariate associations between salient dimensions of trait impulsivity and food reward outcomes at baseline. McDonald’s Omega coefficients were calculated to evaluate the internal reliability of questionnaire subscales (Trizano-Hermosilla & Alvarado, 2016). Continuous data were expressed as means and standard errors unless otherwise stated. Distributions were checked for normality using the Shapiro–Wilk test. *P*-values from inferential tests were two-tailed and set at of 5% as the threshold of statistical significance. Partial *η*^2^ was used as an estimate of effect size for ANOVAs. All analyses were conducted using SPSS v26.

## Results

### Participants

During the active recruitment phase, 34 eligible individuals expressed interest, 5 of whom dropped out prior to their first lab visit due to time constraints or capacity to undertake the entire study protocol. The remaining 29 participants attended the baseline session. A further 4 participants withdrew from the study before completing both intervention and control app-based sessions, thus a final sample of 25 was included in primary analyses (see Table 1). Univariate ANOVAs were conducted comparing means on all demographics and trait appetitive scales between the final sample (*n* = 25) and participants who dropped out after baseline (*n* = 4). Analyses revealed that all mean differences were not statistically significant (all *p*s > 0.05).

**Table 1:** Participant characteristics at baseline

	*M (SD)*	Range
Age	32.36 (5.48)	26–45
Sex (% Female)	68	N/A
AEBQ -Trait Food Responsiveness	3.19 (0.68)	2–4.5
TFEQ – Cognitive Restraint	14.00 (3.16)	6–20
TFEQ – Uncontrolled eating	18.52 (4.09)	13–30
TFEQ – Emotional eating	5.60 (2.12)	3–10
BIS – Attentional	17.48 (3.81)	9–28
BIS – Motor	23.32 (3.93)	17–33
BIS – Non-planning	23.12 (4.29)	16–33
State Hunger	49.76 (25.75)	3–92
State Fullness	36.12 (25.56)	1–99
State Desire to Eat	51.40 (25.52)	5–99
State Thirst	47.00 (28.33)	1–98
State Contentedness	60.28 (18.14)	36–100
State Alertness LFPQ Explicit Liking Fat Appeal Bias LFPQ Explicit Wanting Fat Appeal Bias	61.24 (23.34) 3.47 (21.41) 2.29 (21.06)	1–100 − 36.88–50.75 − 40.75–50.75
LFPQ Implicit Wanting Fat Appeal Bias LFPQ Frequency of Choice Fat Appeal Bias	7.06 (36.52) 2.24 (13.09)	− 62.91–65.53 − 25–24

*N* = 25. AEBQ refers to Adult Eating Behaviour Questionnaire. TFEQ refers to Three Factor Eating Questionnaire R-18. BIS refers to Barratt Impulsiveness Scale. FWA refers to Frequency-weighted Algorithm. LFPQ refers to Leeds Food Preference Questionnaire. FAB refers to fat appeal bias

Generally, state hunger was lower on average during each session than those typically found in lab studies using visual analogue scales to assess hunger in fasted subjects (Gibbons et al., 2014). Although subjective ratings of hunger were slightly lower before the intervention session (*M* = 43.44, *SD* = 30.44) than before baseline (*M* = 49.76, *SD* = 25.75) and control sessions (*M* = 50.88, *SD* = 26.68), the variance across these sessions was not statistically significant, *F*(2, 48) = 0.86, *p* = 0.431, *ηp*^*2*^ = 0.03. Moreover, no significant differences were found for desire to eat across sessions, *F*(2, 48) = 1.12, *p* = 0.334, *ηp*^*2*^ = 0.05. Analyses of pairwise comparisons yielded no significant differences between subjective hunger and desire to eat metrics across all sessions (all *p*s > 0.40).

### Leeds food preference questionnaire

#### Explicit liking and wanting

**Fig. 1:**
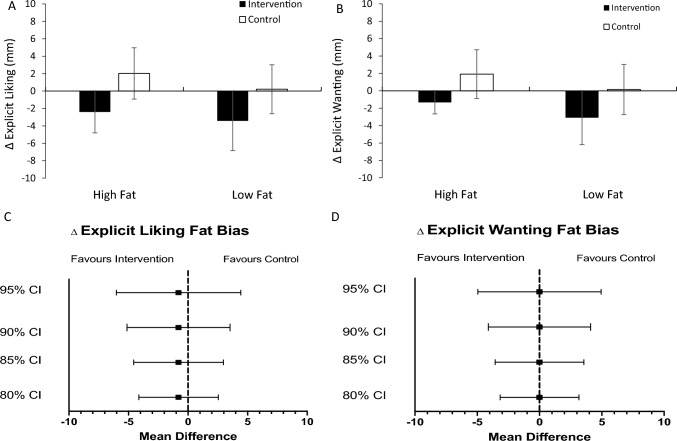
*N* = 25. Changes in explicit liking **A** and explicit wanting **B** from baseline by app-based training task and fat content (i.e., energy density). Errors bars represent the standard error of the mean. MM = millimetres. Panels **C** and **D** depict the mean differences of changes in explicit liking and wanting fat bias, respectively, between app-based training sessions at different confidence limits

A 2-way repeated measures ANOVA of changes in explicit liking for food stimuli based on energy density yielded a statistically significant main effect of app-based activity, *F(*1, 24) = 4.65, *p* = 0.041, *ηp*^*2*^ = 0.16 (Fig. 1). For both high and low fat food stimuli, decreases were observed from baseline to post-intervention activity, whilst an increase or little change was observed from baseline to post-control activity, respectively. The app x fat interaction was non-significant (*F*(1, 24) = 0.10, *p* = 0.756, *ηp*^*2*^ = 0.00), indicating no effect of app on preference for energy-dense foods. The mean difference of changes in explicit liking fat bias between app-based training activities did not indicate a difference between app sessions at any confidence estimate (*M*_Δ_ = -0.80, 95% CI: -6.13, 4.54; Fig. 1).

The 2-way repeated measures ANOVA incorporating ratings of explicit wanting for high and low-fat foods suggested a similar trend to explicit liking, although this main effect did not reach statistical significance (*F*(1, 24) = 2.68, *p* = 0.114, *ηp*^*2*^ = 0.10). In line with explicit liking, no app x fat interaction was observed, *F*(1, 24) = 0.00, *p* = 0.998, *ηp*^*2*^ = 0.00). The lack of variance in energy-dense food preference between training sessions was corroborated by wide confidence estimates for the mean difference (*M*_Δ_ = -0.01, 95% CI: -5.07, 5.06). The mean explicit liking and wanting scores for all food categories were relatively low for every session compared to previous validation studies (i.e., *M* < 45; e.g., Dalton & Finlayson, 2014), which may be commensurate with the low subjective hunger ratings prior to commencing each session.

#### Food choice and implicit wanting

**Fig. 2:**
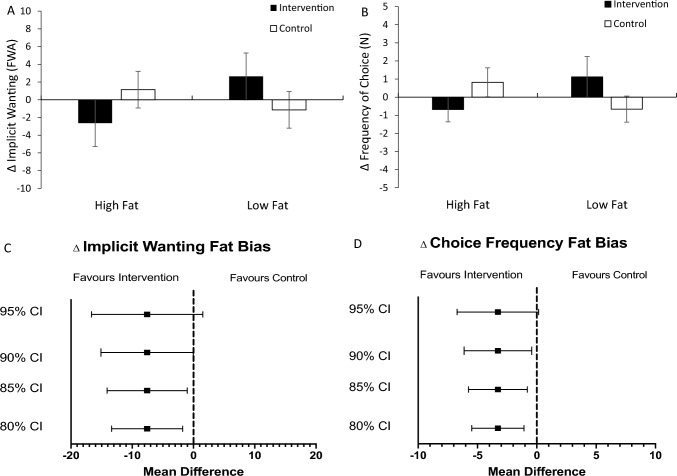
*N* = 25. Changes in implicit wanting **A** and choice frequency **B** from baseline by app-based training session and fat content (i.e., energy density). Errors bars represent the standard error of the mean. FWA = frequency-weighted algorithm. Panels **C** and **D** depict the mean differences of changes in implicit wanting (FWA) and choice frequency fat bias, respectively, between app-based training sessions at different confidence limits

Contrary to results from explicit outcomes, a 2-way repeated measures ANOVA of changes in choice frequency yielded a non-significant main effect of app, *F*(1, 24) = 0.28, *p* = 0.601, *ηp*^*2*^ = 0.06. Rather, relative to control, the intervention reduced selection for high fat foods and commensurately increased selection for low fat foods, *F*(1, 24) = 3.87, *p* = 0.061, *ηp*^*2*^ = 0.14. The mean difference in choice frequency fat bias scores indicated a marginal difference between app sessions at a 10% type 1 error rate (*M*_Δ_ = -3.28, 90% CI: -6.19, -0.38), but not 5% (95% CI: -6.79, 0.23; Fig. 2).

The 2-way repeated measures ANOVA of implicit wanting for foods yielded a non-significant main effect of app (*F*(1, 24) = 0.55, *p* = 0.465, *ηp*^*2*^ = 0.02). Trends similar to those observed in choice frequency was evident, such that a reduction in implicit wanting for high fat foods was observed concurrently with an increase for low fat foods during the intervention session, although the effect size was diminished by comparison (*F*(1, 24) = 2.96, *p* = 0.098, *ηp*^*2*^ = 0.11; Fig. 2). Unlike choice frequency, the mean difference of changes in implicit wanting for energy-dense foods suggested no marginal difference at the 10% error rate (*M*_Δ_ = -7.57, 90% CI: -15.17, 0.04), nor at 5% (95% CI: -16.75, 1.61).

### Exploratory associations: baseline trait impulsivity and food reward

Data were available for *N* = 29 at baseline. A correlation matrix depicting the associations between explicit and implicit fat bias and trait impulsivity scores at baseline are displayed in Table 2. Analyses indicated that Food Responsiveness was consistently and positively associated with all fat bias scores at baseline. Consistent with this finding, Uncontrolled Eating was also positively, albeit not as strongly, correlated with all food reward outcomes. Notably, trait general motor impulsivity was negatively correlated with both explicit and implicit food reward outcomes. No other notable associations were suggested from the computed confidence limits.

**Table 2:** Exploratory Pearson’s correlation coefficients and 95% confidence intervals for associations between mean trait impulsivity scales and LFPQ fat bias scores at baseline

	LFPQ – Explicit Liking	LFPQ – Explicit Wanting	LFPQ- Implicit Wanting	LFPQ – Choice Frequency
AEBQ—Food Responsiveness	.46 [.11, .70]	.44 [.09, .70]	.42 [.06, .68]	.45 [.10, .70]
TFEQ—ognitive Restraint	.10 [− .28, .45]	.08 [− .30, .43]	.03 [− .34, .39]	.05 [− .32, .41]
TFEQ—Uncontrolled eating	.22 [− .16, .54]	.24 [− .14, .56]	.24 [− .14, .56]	.27 [− .11, .58]
TFEQ—Emotional eating	.17 [− .21, .50]	.11 [− .27, .45]	− .06 [− .41, .56]	− .07 [− .42, .31]
BIS—Attentional	− .04 [− .40, .33]	− .07 [− .43, .31]	.15 [− .23, .49]	.13 [− .25, .47]
BIS—Motor	− .50 [− .73, − .16]	− .50 [− .73, − .16]	− .35 [− .64, .02]	− .35 [− .64, − .02]
BIS—Nonplanning	− .15 [− .49, .23]	− .15 [− .49, .23]	− .18 [− .51, .20]	− .20 [− .52, .19]

*N* = 29. *AEBQ* refers to Adult Eating Behaviour Questionnaire. *TFEQ* refers to Three Factor Eating Questionnaire. *BIS* refers to the Barratt Impulsiveness Scale. *LFPQ* refers to the Leeds Food Preference Questionnaire. 95% confidence intervals are uncorrected for multiple comparisons

### Mobile app user experience: Food Trainer

Scores from 26 participants were recorded for the quality assessment of ‘FoodTrainer’ and fell within a range between 1 and 5 for each dimension. ‘FoodTrainer’ was rated favourably on key dimensions of health app quality including its Functionality (*M* = 4.47, *SD* = 0.53), Aesthetics (*M* = 3.77, *SD* = 0.78), and Information (*M* = 3.84, *SD* = 0.77), but only modestly for Engagement (*M* = 2.72, *SD* = 0.81). Overall, a composite based on these objective qualities yielded a moderately high Objective App Quality score on average (*M* = 3.70, *SD* = 0.62). By contrast, the Subjective App Quality score was not rated as favourably (*M* = 2.08, *SD* = 0.82).

## Discussion

In this crossover study, a mobile app-based RIT intervention was tested for its concurrent effects on explicit and implicit facets of food reward. Associations between food-specific and trait impulsivity scales and food reward outcomes at baseline were also evaluated. Analyses indicated that empirical patterns in explicit liking and implicit wanting after RIT appeared to differ in a model where stimulus devaluation of non-specific (i.e., novel) food stimuli was measured. Specifically, trends found in implicit outcomes were discordant from those found in both explicit liking and wanting. Rather, reductions in explicit liking for both energy-dense and low calorie foods during the intervention were marginally significant relative to the control session. Explicit and implicit preferences for energy-dense foods at baseline were generally associated with responsiveness to food cues on average. Additionally, the app-based RIT task was rated favourably on key dimensions of intervention quality, suggesting at least a moderate level of acceptability. Overall, the results suggest that this study design is feasible, and the modality of food reward assessment may be important when testing any generalised effect of RIT. As a feasibility study, reliable conclusions cannot be drawn from the inferential tests and emphasis should be maintained on the descriptive statistics provided.

The utilisation of Berridge and Robinson’s (2016) framework to measure and predict eating behaviours in humans has been scrutinised in the literature. Although it is beyond the scope of this study to elucidate in detail, the interested reader may wish to read critical reviews by Pool et al. (2016), Polk et al. (2017), and Bickel et al. (2018). It has been argued that explicit liking, when measured similarly to the approach in this study, may not be capturing the same appetitive feedback as demonstrated in the animal models conducted by Berridge and Robinson (2016), where liking was measured during food consumption. Indeed, explicit liking and implicit wanting tend to be highly correlated in samples representative of the general population as each measure likely captures an expectation of reward to some degree (Oustric et al., 2020). However, divergences in liking and wanting have been demonstrated in human experiments under particular conditions such as obesity and other eating-related pathologies (Finlayson et al., 2007b, 2011; Morales & Berridge, 2020). Another pertinent question in the context of the present study is whether this framework could provide a utility for assessment of dietary intervention efficacy. In their meta-analysis, Yang and colleagues (2022) only found an effect of RIT when devaluation was measured explicitly, although the number of studies assessing implicit devaluation was far smaller. Although this cannot be equated to a comparison of explicit liking and implicit wanting, it may be of interest for future studies to include both types of evaluations so that coherent trends may be collated and examined in relation to observable eating behaviour such as food selection and intake.

Two notable discrepancies were detected in the observations of this study. First, the main difference between explicit liking and implicit wanting was observed in low-calorie food evaluations specifically, with a decrease being observed in the former in contrast to previous RIT trials (e.g., Lawrence et al., 2015b). Second, trait motor impulsivity as assessed by the BIS was inversely associated with higher explicit and implicit food reward. Such unexpected observations highlight the challenges of appropriately executing the proposed study design. For example, associations between trait impulsivity scales and food reward, and RIT effects, may be state-dependent, and this sample did not demonstrate the same degree of fasting hunger sensations typically observed in ostensibly fasted subjects (e.g., Dalton & Finlayson, 2014). Moreover, the choice of food stimuli and how they are categorised may be conditional factors when observing potential effect generalisation and may additionally rely on pre-existing distinctions held by the individual (Serfas et al., 2017). This likely introduces more error variance, and to the extent that such general effects actually exist, studies with more statistical power would likely be needed to detect them relative to effects on ‘trained’ food stimuli.

There are notable limitations in this study, thus conclusions should be drawn with caution. First, this study had a modest sample size, which may suggest an elevated probability of a type 2 error in analyses as well as overestimated effect sizes (Dechartres et al., 2013). The wide confidence intervals produced are indicative of this fact. However, the contrast in effect sizes between explicit and implicit food reward measures suggests further investigation in a more adequately powered study may be warranted. Although the length of the washout period was standardised, it is uncertain how long effects from RIT are sustained, especially from a single session. Chen and colleagues (2019) demonstrated that changes in food preference were sustained after 1 week after a single training session, albeit with a significantly reduced effect size (also see Adams et al., 2021). Future studies that utilise a repeated-measures design ought to be mindful of these results when designating washout periods of adequate length to mitigate potential carryover effects. No training performance data were available, therefore, adequate learning of stimulus–response associations by each participant cannot be demonstrated, as is standard practice in RIT trials. Indeed, a meta-analysis of RIT interventional studies by Jones and colleagues (2016) found that accuracy on inhibition trials (i.e., commission error rate) was a significant predictor of RIT efficacy to modify eating behaviours. It is therefore important that future studies record performance data when discerning between no effect or lack of compliance. Finally, the choice of control comparison did not have energy-dense food cues, which may have influenced differences between sessions independent of the training mechanism. Future studies ought to utilise different types of control tasks to provide more confidence in the reliability of these results.

In conclusion, this proof of concept study provided preliminary evidence for the feasibility of applying Berridge and Robinson’s (2016) Incentive Sensitisation framework for assessing the efficacy of RIT to modulate appetite. Observations suggest that the LFPQ may be associated to the food responsiveness dimension of trait impulsivity at baseline. It is thus proposed that the LFPQ can be a suitable and valid instrument to assess efficacy of behavioural interventions to modify food hedonics. Effect generalisation appears to be feasible, but this may be more apparent when evaluations are measured as implicit wanting. Adequately powered, pre-registered trials are needed to reproduce these observations and infer any relationships with confidence and further examine how salient factors, such as trait impulsivity or food stimulus specificity, may moderate the RIT effect on explicit liking or implicit wanting for palatable, high-energy foods concurrently. Additionally, studies may measure liking and wanting for both trained and novel food stimuli in order make direct comparisons of these facets of reward based on stimulus specificity.

### Electronic supplementary material

Below is the link to the electronic supplementary material.

Supplementary file1 (DOCX 2029 KB)

## Data Availability

The authors have custody of the complete de-identified dataset, and it is available on the QUT Research Data Finder repository under the corresponding author’s name and keywords of this manuscript (https://researchdatafinder.qut.edu.au/).

## Footnotes

1 Participants were informed during recruitment that they would be testing two mobile health apps designed to promote healthier eating habits, which served to conceal the nature of the study and thus reduce response bias. However, awareness of study purpose was not checked upon completion.
