# Supplementary material for: Response inhibition training as an intervention to modify liking and wanting for foods based on energy density: a proof of concept study
Source: J Behav Med. 2023 Oct 9;47(2):271–81. doi: 10.1007/s10865-023-00453-3 (PMC10944450; doi:10.1007/s10865-023-00453-3)
Supplement: Supplementary file 1 — Supplementary file1 (DOCX 2029 KB) [file 10865_2023_453_MOESM1_ESM.docx]

***A1. Supplementary data***

*A1.1. Leeds Food Preference Questionnaire (Finlayson et al., 2007a)*

*A1.1.1. Single Food Task (Oustric et al., 2020)*


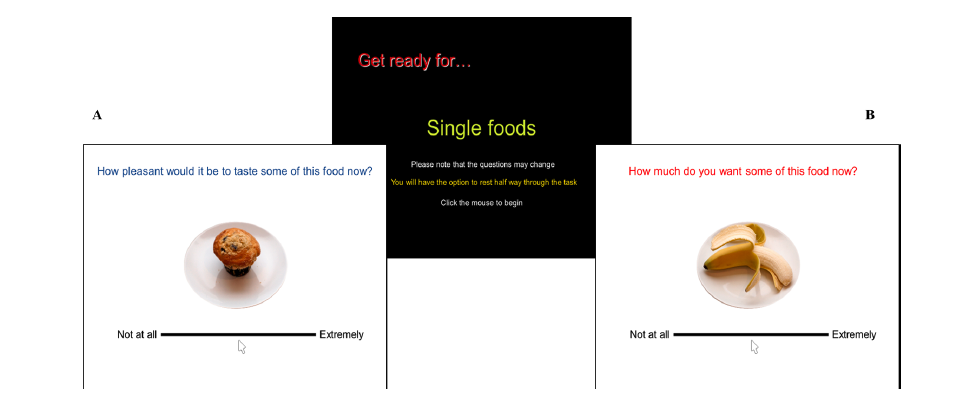


**Figure A1.1.** A graphical depiction of the single food task from the LFPQ. Panel A is an example of the Explicit Liking assessment. Panel B is an example of the Explicit Wanting assessment.

*A1.1.2. Paired Food Task (Oustric et al., 2020)*


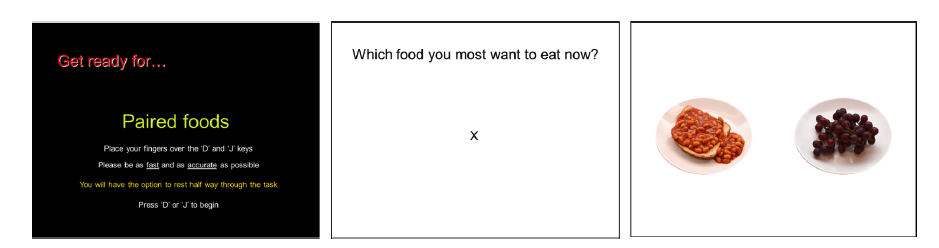


**Figure A1.2.** A graphical depiction of the paired food task from the LFPQ. Participants are presented with two foods and asked to select one as quickly and as accurately as possible. Reaction time to selection is covertly recorded.

*A1.2. ‘FoodTrainer’ - Response Inhibition Training Mobile Application (University of Exeter, 2017)*


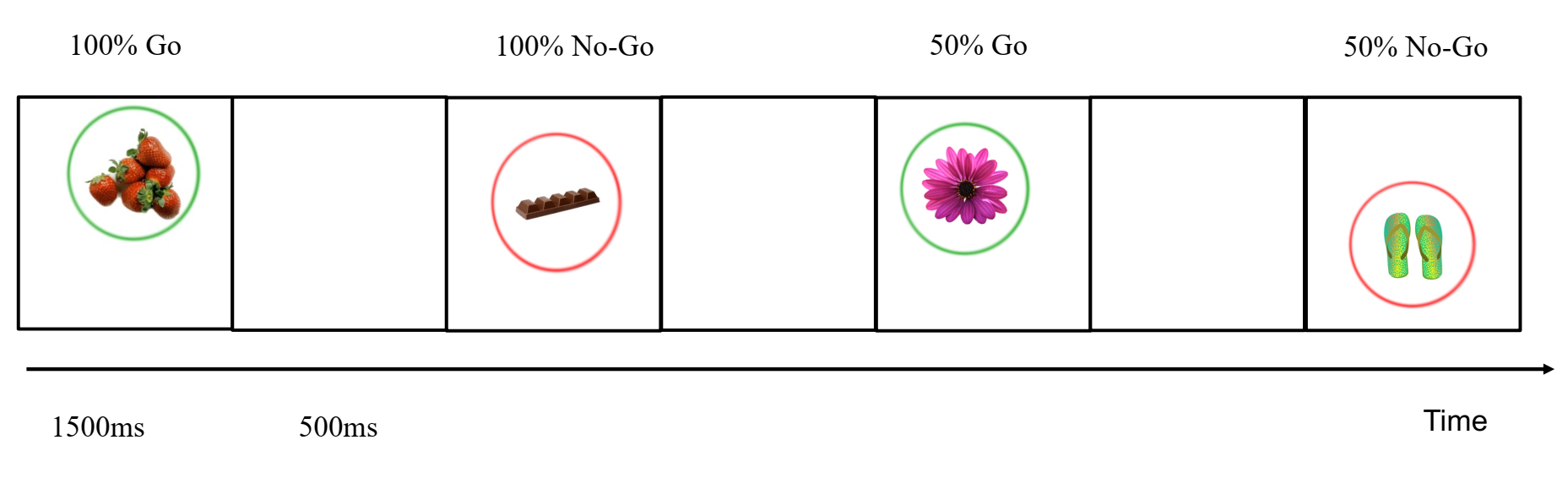


**Figure A1.3.** Schematic illustration from the ‘FoodTrainer’ RIT task with both food and neutral stimulus types. Red circles denote visual cue for response inhibition and green circles denote cues for approach. ‘Healthy’ food stimuli always required an approach where the user had to press the centre of the green circle and energy-dense stimuli always required response inhibition. Neutral stimuli consisted of flowers or household items and were equally likely to require approach or response inhibition.

*A1.3. User Mobile Application Rating Scale Results (Stoyanov et al., 2016)*

**Figure A1.4.** *N* = 26; Mean objective and subjective evaluations of app quality from the User Mobile Application Rating Scale (uMARS) for ‘FoodTrainer’ intervention app. Errors bars represent the standard error of the mean.
